# Prevalence of Neurolathyrism and its associated factors in Grass pea cultivation areas of Dawunt District, North-eastern Ethiopia; 2022: a community based multilevel analysis

**DOI:** 10.1186/s12883-023-03379-0

**Published:** 2023-10-05

**Authors:** Melaku Bimerew, Teshome Gebremeskel, Biruk Beletew, Manay Ayalneh, Wondye Ayaliew, Mulugeta Wodaje

**Affiliations:** 1 Department of Pediatric and Child Health Nursing, College of Medicine and Health Science, Injibara University, Injibara, Ethiopia; 2 Department of Human Anatomy, College of Health Science, Woldia University, Woldia, Ethiopia; 3 Department of Nursing, College of Health Science, Woldia University, Woldia, Ethiopia; 4 Department of Plant Biotechnology, Woldia University, Woldia, Ethiopia; 5 Department of Midwifery, College of Health Science, Woldia University, Woldia, Ethiopia

**Keywords:** Neurolathyrism, Prevalence, Dawunt, Ethiopia

## Abstract

**Introduction:**

: Neurolathyrism is an upper motor neuron disorder characterized by spastic paraparesis, which is caused by the prolonged over-consumption of grass pea. It is a devastating disease with great impacts on physical, social, mental, and economical health.

**Objective:**

To determine the prevalence of neurolathyrism and its associated factors in grass pea cultivation areas of Dawunt wereda.

**Methods:**

Community based cross-sectional study design was conducted from February 01- March 30, 2021 on 631 Households with a total of 3,350 individuals. Two-stage random sampling technique was used to select participants. Multilevel binary logistic regression was used to identify factors associated with neurolathyrism. Statistical significance was declared at p < 0.05; and AOR with 95% CI was used to interpret the results.

**Results:**

The household and population level prevalence of neurolathyrism in Dawunt district were 9.2% (7.2–11.7%) and 2.4% (2.0-2.3.0%) respectively. Age (AOR = 7.4 ( 2.6–20.6)), male sex (AOR = 7.8 (3.9, 15.4)), and marital status (AOR = 4.0 (1.3–12.8)) were the individual level variables; family size (AOR = 12.6 (3.0-52.8)), annual grass pea production (AOR = 5.0 (2.3–11.0)**)**, ever feeding only grass pea (AOR = 8.8(3.5–22.2)), ever feeding immature seeds of grass pea (AOR = 6.28 (2.80, 14.08)), high grass pea to other cereals mixing ratio (> 3:1) (AOR = 6.1 (1.1, 33.5)) were the household level variables found to have significant association with neurolathyrism.

**Conclusion:**

The prevalence of neurolathyrism was found to be high. Ever feeding only grass pea, Grass pea to other cereals mixing ratio (using ratio of 1:1 or more), and Ever feeding immature grass pea seeds were the modifiable risk factors for neurolathyrism.

## Introduction

Lathyrism is a chronic and irreversible medical condition caused by overconsumption of certain legumes of the genus Lathyrus [1]. There are three types of lathyrism; namely:- Osteolathyrism—a disease caused by ingestion of Lathyrus odoratus (sweat pea) seeds containing beta-aminopropionitrile toxin affecting the bones and connective tissues [2, 3]; Angiolathyrism—a disease caused by the same toxin causing osteolathyrism; but affecting the blood vessels [4]; and Neurolathyrism—a disease commonly caused by ingestion of Lathyrus sativus (grass pea) seeds containing oxalyldiaminopropionic acid (ODAP) toxin affecting the motor neurons; all of which results in permanent and irreversible disability [5, 6].

In fact, sporadic ingestion of grass pea might not cause neurolathyrism; and simple measures like boiling and prolonged soaking are believed to decrease the toxic effects of grass pea by as high as 90% [1, 7, 8]. But consuming too much of grass pea (more than one-third of one’s daily dietary intake) continuously for more than two or three months without taking measures to reduce its toxic effect is believed to cause accumulation of ODAP [5]. ODAP accumulation in turn causes excitotoxicity and oxidative stress mediated degeneration of the pyramidal tract neurons (upper motor neurons) in the spinal cord and in the area of the cortex controlling the legs. This degeneration will end up with spastic paraparesis—a major clinical phenomenon of neurolathyrism [9–11].

The paraparesis in neurolathyrism may follow acute or insidious onset [12, 13]. After onset it will continue throughout life; having four major stages: Stage-I—mild disability with no need of sticks; Stage-II—moderate disability requiring support with one stick; Stage-III—severe disability requiring support with two sticks; and Stage-IV—very severe disability with inability to move with supporting sticks (crawler stage) [14]. Unfortunately, once the paraparesis occurs, neither treatment nor discontinuation of grass pea consumption can reverse the disability; but discontinuation can limit or slow its progress [15].

Since grass pea is a drought-tolerant and high yielding legume, it is more cultivated and consumed in areas of the world where there is recurrent drought; as in Bangladesh, India, Nepal, and many parts of Africa. Grass-pea is extensively cultivated in the highlands of north and central Ethiopia, including in Dawunt wereda. Hence, neurolathyrism is a major public health issue in those countries. Literature indicates that outbreaks of neurolathyrism have been reported in the countries of Indian sub-continent, Europe, China, Afghanistan, and North Africa [16, 17]. Among other countries of the world, neurolathyrism has been an endemic disease in Ethiopia, India, and Bangladesh [5, 18]; with periods of outbreak in times of famine. A neurolathyrism prevalence of as high as 2.9% has been reported in Ethiopia.^19^

Neurolathyrism is a devastating disease which needs to be classified as a reportable disease as it occurs in endemics; resulting in irreversible spastic myelopathy [18]. It has a great impact on health of a human being by causing permanent disability. This in turn increases the burden of health care systems; and might increase health care expenditures. Besides it commonly affects the young males who are the main working forces for a country. Permanent disability of those working forces in turn has a great impact on economic growths. Furthermore, it has great psychosocial impact on the victims; causing lack of marriage, increased divorce rates, occupation choices, depression, social isolation, unplanned migration, and family separation [19–21].

Due to personal disability and subsequent socioeconomic effects, neurolathyrism deserves a great attention. Previously, a limited number of studies from India, Bangladesh, and Ethiopia had been revealed the prevalence of neurolathyrism [18, 22, 23]. But, time has been elapsed and currently neurolathyrism is considered as a disease of the past; with very rare new cases. Hence, despite its grave consequences, currently the disease has not received due concern from the public, health authorities, and the scientific community; especially in Ethiopia. Factors associated with neurolathyrism have not been addressed well in Dawunt wereda, Ethiopia. Furthermore, timely updating of the prevalence of neurolathyrism will add a value on the existing knowledge, and give insight for policy makers, project designers, and other concerned bodies. Therefore, the purpose of this study was to determine the prevalence and associated factors of neurolathyrism among inhabitants of Dawunt wereda, North Wollo Zone, Northeastern Ethiopia.

## Methods

### Study design, area, and period

This was a community based cross-sectional study conducted in Dawunt district, North Wollo Zone, Amhara region, North-eastern Ethiopia; where cultivation of grass pea is high. The district is divided in to 15 administrative kebeles; and had an estimated population of 80, 211 in 18,653 households. Of the 15 kebeles, 11 kebeles are grass pea cultivation areas [24]. Therefore, this study was conducted in those kebeles from February 01 to March 30/ 2021.

### Eligibility criteria

All households and/ or individuals who have lived at least 1 year in grass pea cultivation areas of Dawunt district were included in the study; while individuals whose age is less than 1 year (infants) were excluded from the study.

### Sample size and sampling technique

A total of 635 households, with a total population of 3,350 were used to address the objectives of this study. Two-stage random sampling technique was used to select the samples. 1st stage: By considering the rule of thumb, 4 kebeles were selected randomly from the total 11 grass pea cultivating kebeles with lottery method. 2nd stage: By using proportional allocation and systematic random sampling technique (sampling interval (K) = 8), 635 households were selected from the total households of the 4 kebeles (4,964 households) selected in the 1st stage.

### Variables

#### Dependent variable

- Prevalence of neurolathyrism.

### Independent variables

#### • level 1 (individual level variables)

- Age, Sex, Marital status, Educational status, and Occupation.

#### • level 2 (Household level variables)

- Family size, Average monthly income, Having a farming land, Main income source, Annual Grass pea production amount, Ever feeding grass pea only, Usual cooking utensils, Grass pea to other cereals mixing ratio, Ever feed grass pea as immature seeds, Ever feed as roasted seeds.

### Data collection methods, tools, and procedure

Semi-structured interviewer administered questionnaire adapted from peer reviewed literatures [15, 17–19, 25] was used to collect the data by using face to face interview and clinical diagnosis. The interview was conducted in local (Amharic) language by 8 BSc nurses; and the clinical diagnosis of neurolathyrism was made by 2 physicians. Data collection was supervised by two public health professionals who have a master’s degree.

### Criteria for clinical diagnosis of Neurolathyrism

Neurolathyrism was diagnosed in patients with symmetrical spastic paralysis of lower extremities with intact sensory perception AND walking on the balls of the feet with lurching and scissoring type gait AND sign/ symptom begins after and during consumption of grass pea AND clinical exclusion of other causes of paralysis (14, 26).

### Data quality control

For insuring data quality, training was given for data collectors and supervisors about the purpose of the study, the questionnaire in detail, and the data collection procedure. Pre-test of the questionnaire was conducted on 32 HHs in Delanta; and necessary modifications were made accordingly. Besides, the completeness, accuracy, and consistency of the data were checked daily by data collectors. Supervisors & investigators were followed the data collection process closely. Moreover, coding, careful data entry, and cross-tabulation were used.

### Data analysis and presentation

Data were entered using EPI-data Version 4.2.0 by clustering individual level variables with in a household. Then, data were exported to STATA Version 16.0 for analysis. Descriptive statistics was employed to describe individual level and household level characteristics of the study participants. Multilevel Binary logistic regression analysis was used to assess the association between dependent and independent variables. In this multilevel logistic regression, four models were employed.

The first model was the null model (Model 1) containing no independent variables; and it was used to check the need for clustering (multilevel analysis) by checking variability of neurolathyrism among the households. An ICC value of ≥ 5% was used to declare significant variability in neurolathyrism among the (clusters) households.

The second and third models were Model 2 and Model 3 which were used to identify significant individual and household level variables respectively. Variables with a p-value of < 0.25 in both models were considered as significant; and were candidates for the final model.

The final model was Model 4 in which both individual level and household level variables entered together to identify statistically significant factors associated with neurolathyrism. Multi-collinearity between independent variables was cheeked by correlation matrix; and model fitness was checked by AIC and BIC. Statistically significant association was declared at a p-value of < 0.05; and AOR with 95% CI was used to interpret the association.

## Results

### Sociodemographic characteristics of the study participants

A total of 631 households, with a total population of 3350, were interviewed; giving a response rate of 99.4%. Four households with an estimated number of 21 (0.6%) individuals were non-respondents. The minimum and maximum age of the participants was 1year and 85 years respectively with a mean ± SD of 25.0 ± 15.4years. About 57% of the participants were adults (aged from 18-64years). The male to female ratio of the study participants was 1:1.07; with 51.6% of the participants being females. Most of the participants (40.6%) had not taken formal education; about 61% had not ever married; and about 36% are farmers (Table 1).

**Table 1:** Sociodemographic (individual level and household level) characteristics of the study participants in Dawunt wereda, northeastern Ethiopia; 2021

Variables			Frequency (n)	Percentage (%)
**Individual level variables (N = 3,350)**				
Age group	Children (1-17years)		1408	42.0
	Adults (18-64years)		1897	56.6
	Geriatrics (65^+^years)		45	1.3
Sex	Male		1620	48.4
	Female		1730	51.6
Educational status	No formal education		1361	40.6
	Primary		1307	39.0
	Secondary and above		682	20.4
Marital status	Married		1241	37.0
	Divorced		38	1.1
	Widowed		33	1.0
	Not ever married		2038	60.8
Occupation	Occupied (Farmer)		1195	35.7
	Not occupied		435	13.0
	Occupied (Non-farmer)		1720	51.3
**Household level variables (N = 631)**				
Family size		< 5	203	32.2
		5–7	364	57.7
		> 7	64	10.1
Average monthly income		< 1000 ETB	508	80.5
		1000–2000 ETB	72	11.4
		> 2000 ETB	51	8.1
Main income source		Farming	596	94.5
		Non-farming (trading, employment)	35	5.5
Farming land		Yes	612	97.0
		No	19	3.0
Average annual grass pea production		< 6quintals (below the mean)	466	73.9
		6 quintals and above (mean and above)	165	26.1
Family ever feed grass pea only		Yes	42	6.7
		No	589	93.3
Main cooking utensils		Clay materials	505	80.0
		Non-clay materials	126	20.0
Grass pea to other cereals mixing ratio		< 1:1	597	94.6
		1:1 to 3:1	27	4.3
		> 3:1	7	1.1
Family ever feed roasted grass pea		Yes	69	10.9
		No	562	89.1
Family ever feed grass pea as immature seeds		Yes	98	15.5
		No	533	84.5

Of the total 631 households, about 58% had 5–7 family members; about 81% has an average monthly income of < 1000ETB; and farming was the main source of income for almost all (96%) of the households. About 97% of the households have their own farming land; and all of the participants cultivate grass pea annually with a mean annual grass pea production of 6 quintals. Most of the households (74%) produce < 6 quintals of grass pea per year. About 7% of the households (n = 631) had ever fed “only grass pea” for a minimum of 1month and a maximum of 8months; with a mean ± SD of 2.8 ± 1.8months; and all of the households who had ever fed only grass pea for 5-8months had at least one neurolathyrism patient with in them. Based on the individual level analysis, about 8.3% (n = 206) and 38.2% (n = 34)of the individuals who had ever fed only grass pea for 1–4 months and 5-8months were found to have neurolathyrism. All households had consumed grass pea by mixing it with wheat; and about 95% of the households mix grass pea with wheat in less than 1:1 ratio. Moreover, About 11% and 16% of the households had consumed grass pea by roasting and as immature seeds respectively (Table 1).

### Prevalence of Neurolathyrism and characteristics of the cases

The household level and the population level prevalence of neurolathyrism in Dawunt district was 58 (9.2%; 95% CI = 7.2–11.7%; n = 631households) and 81 (2.4%; 95% CI = 2.0–3.0%; n = 3,350 individuals) respectively. Sex specific analysis showed that the prevalence of neurolathyrism to be 4.1% (n = 1,620) in males and 0.9% (n = 1,730) in females. Based on age specific analysis, the prevalence of neurolathyrism was found to be 8.3% (n = 60), 4.2% (n = 650), 4.0% (n = 202), 2.9% (n = 484), 2.7% (n = 337), and 1.5% (n = 1,233) among individuals aged 60-69years, 20-29years, 50-59years, 40-49years, 30-39years, and 10-19years respectively. Neurolathyrism cases were not found among individuals aged from 1-9years (n = 365) and individuals aged 70years and above (n = 19).

A minimum of zero and a maximum of 4 neurolathyrism cases were documented in each household with neurolathyrism. Individuals with Neurolathyrism were aged from 14-65years; with a mean ± SD of 32.1 ± 14.4years. The age of onset for neurolathyrism was ranged from 4-37years; with a mean ± SD of 15.8 ± 7.0years. Most of the individuals (82%) with neurolathyrism were males; with a male to female ratio of 4.4:1. About 46% and 42% of the individuals with neurolathyrism were at stage-I and stage-II neurolathyrism paralysis respectively (Table 2).

**Table 2:** Frequency distribution of neurolathyrism cases per household and Characteristics of the individuals with Neurolathyrism in Dawunt wereda; North Wollo Zone, Northeastern Ethiopia; 2021

Variables		Frequency (n)	Percentage (%)
Number of Neurolathyrism cases per household (N = 58)	1 case	40	69.0
	2 cases	14	24.1
	3 cases	3	5.2
	4 cases	1	1.7
Sex of cases (N = 81)	Male	66	81.5
	Female	15	18.5
Current Age of cases (N = 81)	1–17	11	13.6
	18–64	69	85.2
	65^+^	1	1.2
Neurolathyrism/ paralysis onset age (N = 81)	1-5years	2	2.5
	6-12years	31	38.3
	13-19years	27	33.3
	20-35years	20	24.7
	36 + years	1	1.2
Neurolathyrism stage (N = 81)	Stage-I	37	45.7
	Stage-II	34	42.0
	Stage-III	7	8.6
	Stage-IV	3	3.7

### Factors associated with neurolathyrism

#### Test of Model fitness

Among the four models employed in this study, the final model (Model 4) had been better fitted than others; as AIC of Model 4 (538.545) was less than AIC of model 3, 2, and 1 (607.089, 653.065, and 709.218) respectively (Table 3).

**Table 3:** Multilevel logistic regression analysis of factors associated with Neurolathyrism in Dawunt wereda, North Wollo zone, Northeastern Ethiopia; 2021

Variables		Neurolathyrism		Model-1	Model-2 AOR (95% CI)	Model-3 AOR (95% CI)	Model-4 AOR (95% CI)	P-value (model-4)
		Yes	No					
**Random effects**								
ICC (95% CI)				0.573 (0.435, 0.701)	0.593 (0.431, 0.738)	0.414 (0.269, 0.576)	0.384 (0.227, 0.569)	
PCV				Reference	-8.5%	47.4%	53.6%	
Variance (SE)				4.42 (1.26)	4.80 (1.61)	2.33 (0.77)	2.05 (0.79)	
MOR				11.49	12.74	5.89	5.27	
**Model fitness**								
AIC				709.218	653.065	607.089	538.545	
BIC				721.451	726.466	698.839	679.229	
Log likelihood				-352.609	-314.533	-288.544	-246.272	
**Individual level variables**								
Age group	1-17years	11	1397		1		1	
	18-64years	69	1828		5.39* (1.93, 15.04)		7.38*** **(2.64, 20.61)**	< 0.001
	65^+^years	1	44		1.16 (0.08, 17.14)		1.42 (0.11, 19.02)	0.792
Sex	Male	66	1554		6.54* (3.32, 12.89)		7.76*** **(3.91, 15.40)**	< 0.001
	Female	15	1715		1		1	
Educational status	No formal education	41	1320		2.95* (0.91, 9.59)		3.18 (0.99, 10.23)	0.052
	Primary	25	1282		2.02* (0.78, 5.29)		2.39 (0.93, 6.18)	0.071
	Secondary^+^	15	667		1		1	
Marital status	Married	40	1201		1		1	
	Divorced	3	35		4.76* (0.80, 28.28)		2.05 (0.29, 14.13)	0.468
	Widowed	1	32		1.74 (0.15, 20.38)		0.80 (0.08, 8.59)	0.856
	Not ever married^X^	37	2001		2.99* (0.92, 9.68)		4.02** **(1.26, 12.81)**	**0.018**
Occupation	Farmer	44	1151		1.77 (0.52, 6.02)		2.00 (0.63, 6.34)	0.238
	Not occupied^Y^	3	432		0.32* (0.06, 1.83)		0.27 (0.05, 1.54	0.140
	Non-farmer^Z^	34	1686		1		1	
**Household level variables**								
Family size	< 5	24	673			11.96* (2.93, 48.75)	12.64*** **(3.02, 52.81)**	**0.001**
	5–7	51	2065			3.75* (1.04, 13.49)	3.27 (0.90, 11.85)	0.072
	> 7	6	531			1	1	
Average monthly income	< 1000 ETB	71	2692			3.43* (0.58, 20.30)	3.57 (0.59, 21.66)	0.166
	1000–2000 ETB	8	347			2.69 (0.37, 19.40)	2.75 (0.37, 20.24)	0.320
	> 2000 ETB	2	230			1	1	
Main income source	Farming	80	3161			3.18 (0.13, 78.75)		
	Non-farming	1	108			1		
Farming land	Yes	80	3225			0.51 (0.02, 17.17)		
	No	1	44			1		
Annual grass pea production	< 6quintals	36	2370			1	1	
	6 quintals^+^	45	899			4.66* (2.14, 10.14)	4.99*** **(2.26, 10.99)**	**< 0.001**
Family ever feed grass pea only	Yes	30	210			8.32* (3.29, 21.02)	8.75*** **(3.45, 22.21)**	**< 0.001**
	No	51	3059			1	1	
Main cooking utensils	Clay materials	72	2685			1.88* (0.67, 5.27)	2.04 (0.70, 5.91)	0.190
	Non-clay materials	9	584			1	1	
Grass pea to other cereals mixing ratio	< 1:1	63	3104			1	1	
	1:1 to 3:1	12	132			3.32* (1.03, 10.70)	3.78** **(1.16, 12.28)**	**0.027**
	> 3:1	6	33			5.48* (0.91, 33.02)	6.05** **(1.09, 33.47)**	**0.039**
Ever feeding roasted grass pea	Yes	23	391			2.01* (0.81, 4.99)	2.10 (0.85, 5.16)	0.107
	No	58	2878			1	1	
Ever feeding immature seeds	Yes	47	506			5.38* (2.42, 11.97)	6.28*** **(2.80, 14.08)**	**< 0.001**
	No	34	2810			1	1	

^*X*^ *Includes singles and the non-eligible (Male < 20 years; Female less than 18 years)*

^*Y*^ *Includes non-occupied (if age ≥ 7years) and the non-eligible (Age < 7years)*

^*Z*^ *Includes merchants, government employs, private employs, and students*

*Significant at p-value < 0.25 in Model-2 or Model-3

**Statistically significant at p-value < 0.05 in the final model (Model-4)

***Statistically significant at p-value ≤ 0.001 in the final model (Model-4)

*AIC, Akaike Information Criteria; AOR, Adjusted Odds Ratio; BIC, Bayesian Information Criteria; CI, Confidence Interval; ICC, Intra-cluster Correlation Coefficient; MOR, Median Odds Ratio; PCV, Proportional Change in Variance; SE, Standard Error*

### Test of the need for multilevel analysis

In this multilevel logistic regression, the results of Model 1 showed that there was statistically significant variability in the odds of having neurolathyrism among clusters (households) (ICC = 57.3% (95% CI: 43.5–70.1%)). This means about 57.3% of variability in the odds of having neurolathyrism was attributable to differences in the household level characteristics, while the remaining 42.7% was attributable to individual level characteristics; supporting the need for a multilevel analysis (Table 3).

### Identifying significant individual level variables

In Model 2, five (5) individual level variables were entered. All of those variables were significantly associated with an increased odds of neurolathyrism at a p-value of < 0.25, and were found to be candidates for the final model (Model 4) (Table 3).

### Identifying significant Household level variables

In Model 3, ten (10) Household level variables were entered. In this model, eight (8) variables were found to be significantly associated with an increased odds of neurolathyrism at a p-value of < 0.25, and were found to be candidates for the final model (Model 4) (Table 3).

#### Statistically significant variables associated with neurolathyrism

In the final model (Model 4), a total of 13 variables (5 individual level variables and 8 household level variables) were entered. Of them, eight (8) variables were found to have a statistically significant association with an increased odd of neurolathyrism (Table 3).

Individuals aged from 18 to 64 years were 7.38 times more likely to have neurolathyrism than individuals aged from 1-17years (AOR = 7.38, 95% CI = 2.64–20.61). The odds of having neurolathyrism was also 7.76 times more likely in males than females (AOR = 7.76, 95% CI = 3.91–15.40). Moreover, individuals who had not ever married were 4 times more likely to have neurolathyrism than married individuals (AOR = 4.02, 95% CI = 1.26–12.81) (Table 3).

Individuals living in households whose family members were less than five (< 5) were 12.64 times more likely to have neurolathyrism than living in households with family members greater than seven (> 7) (AOR = 12.64, 95% CI = 3.02–52.81). The odds of having neurolathyrism was also 5 times higher among individuals living in households with an average annual grass pea production amount of greater than six (> 6) quintals than their counterparts (AOR = 4.99, 95% CI = 2.26–10.99**)**. Individuals living in a household whose family members had ever fed only grass pea for some period of time were also 8.75 times more likely to have neurolathyrism than individuals living in a household whose family members had not ever fed only grass pea (AOR = 8.75, 95% CI = 3.45–22.21) (Table 3).

Ever feeding immature seeds of grass pea was found to increase the odds of neurolathyrism by 6.28 folds (AOR = 6.28, 95% CI = 2.80, 14.08). The odds of having neurolathyrism was 6 times higher among individuals taking grass pea by mixing with other cereals with a ratio of > 3:1 than individuals taking grass pea by mixing with other cereals with a ratio of < 1:1 (AOR = 6.05, 95% CI = 1.09, 33.47). Similarly, individuals taking grass pea by mixing with other cereals with a ratio of 1:1 to 3:1 were 3.78 times more likely to have neurolathyrism than individuals taking grass pea by mixing with other cereals with a ratio of < 1:1 (AOR = 3.78, 95% CI = 1.16, 12.28) (Table 3).

## Discussion

This study was conducted in a district of Ethiopia, in which grass pea is cultivated abundantly. It was aimed to determine the prevalence of neurolathyrism and to identify its associated factors. The household level prevalence of neurolathyrism in the district (Dawunt district) was found to be 9.2%. Studies assessing the household level prevalence of neurolathyrism are generally limited; but a study from Debre Sina, Ethiopia [14] had revealed the household level prevalence of neurolathyrism to be 9.5%, which is similar with the finding of this study. This study has also revealed the individual (population) level prevalence of neurolathyrism in Dawunt district to be 2.4%; which was higher than previous studies conducted in Dembia and Fogera (Ethiopia) [25], Legambo (Ethiopia) [18], Bangladesh [22], and India [23]; as those studies revealed a prevalence of 0.6%, 0.25%, 0.14%, and 0.7% respectively. This discrepancy might be due to differences in study areas, period, and sample size. On the other hand, a nearly similar finding (prevalence = 2.38%) was reported from studies conducted in Debre Sina, Ethiopia [14]. This study has also revealed most (88%) of neurolathyrism patients to be at stage-I or II of paralysis; which was similar with findings of previous studies.

Regarding to the onset of neurolathyrism, this study had revealed that the neurolathyrism onset age to be ranged from 4 to 37 years with a mean and standard deviation of 15.7 ± 7.0years. About 38.3%, 33.3%, 24.4%, and 2.5% of the population had a neurolathyrism onset age of 6-12years, 13-19years, 20-35years, and 1-5years respectively. This result implies that the prevalence of neurolathyrism is higher among individuals aged from 6-35years; contributing almost 95% of neurolathyrism prevalence. This result was similar with previous studies [14, 22, 26].

In this study, Males were nearly 8 times more likely to be affected by neurolathyrism than females. Previous studies from Ethiopia [14, 26] and India [23, 26], had also showed that neurolathyrism affected predominately males than females. The reason for this male predominance is not well-known—needing further researches. But one study from Ethiopia had justified that this might be due to the fact that in Ethiopia males are more involved in the agricultural production and harvesting process which might lead them to consume higher amounts of raw unprocessed grass pea seeds than females [27]. Another possible explanation might be higher daily nutrient requirements in males than females. For example, daily requirement for sulphur containing amino acids is higher in males which lead them to have higher risk of sulphur deficiency; and sulphur deficiency was reported as a factor for neurolathyrism [28].

Individuals aged from 18-64years (Adults) were nearly 7 times more likely to have neurolathyrism than individuals aged less than 18years (children). A study from India [23] had also reported that most of patients with neurolathyrism to be aged 20years and above. But findings of this study should be interpreted in caution; it does not imply that neurolathyrism starts (manifests) in individuals aged 18-64years; rather it describes that currently most of the neurolathyrism patients in the study area are adults (aged 18-64years). In this study, the pick age of onset for neurolathyrism was found to be 6-19years, which was similar with previous studies conducted in Ethiopia [14, 26]. Individuals who had not ever married were found 4 times more likely to have neurolathyrism than individuals who are currently married. In a previous study conducted in Ethiopia [14], marital status was not associated with increased odds of neurolathyrism. This discrepancy may be due to differences in study area and period.

Family size (having a family member of < 5), Annual grass pea production amount (producing 6 quintals or more per year), Ever feeding only grass pea, Grass pea to other cereals mixing ratio (using ratio of 1:1 or more), and Ever feeding immature grass pea seeds were the household level variables found to have statistically significant association with an increased odds of neurolathyrism. Individuals living in households with a family member of less than 5 were nearly 13 times more likely to have neurolathyrism than individuals living in households with greater than 7 family members. This was in contradiction with a previous study conducted in Ethiopia [18].

Individuals living in a household with an annual grass pea production of ≥ 6 quintals were 5 times more likely to have neurolathyrism than individuals living in a household with an annual grass pea production of < 6 quintals. Individuals who live in a household that had ever fed only grass pea were also nearly 8 times more likely to have neurolathyrism than their counterparts. Moreover, individuals living in a household that had ever fed grass pea as immature seeds ((Green unripe) or “Eshet” in Amharic) were nearly 6 times more likely to have neurolathyrism than who live in households that did not ever fed as immature seeds. Immature seeds of grass pea are consumed without processing like soaking, boiling, or roasting. So, the toxic components of grass pea is high in immature seeds than the mature seeds; and consuming immature seeds is associated with increased odds of neurolathyrism as revealed by this study and a previous study from Ethiopia [18].

Consuming grass pea by mixing it with other cereals using a high grass pea to other cereals ratio was also found to be associated with increased odds of neurolathyrism. A previous study conducted in Ethiopia had also revealed eating grass pea as a pancake (“Enjera” in Amharic) by mixing with less than one-third cereal (mixing ratio > 3:1) was associated with a 2.28 times increased risk of acquiring neurolathyrism [26].

### Limitation of the study

This study had revealed the prevalence of neurolathyrism and identified individual level and household level factors. Robust research methods were employed. However, this research might not be free from some limitations as some of the associated variables had wide CI, which was due to small number of observed cases with relatively larger sample size. In this study, the estimation for the study population was based on the 2016 population statistics of Dawunt district; which might not indicate the true population statistics of 2021. This study used only clinical-based diagnostic criteria for neurolathyrism without laboratory investigations and imaging studies due to a lack of resources. Authors of this article believe that the possibility of misdiagnosis is very low as an in-depth clinical analysis of differentials was employed. But, future researchers should also consider blood tests and imaging studies to have more advanced diagnostic criteria than this study.

Since this study was a cross-sectional study, it has not revealed the incidence of neurolathyrism in the study area. In fact, incidence data might have a great public health importance than the prevalence data; as it provides the number of new cases over time. Similarly, this study had not assessed if social and climatic factors have an effect on neurolathyrism. Therefore, future researchers should consider assessing the incidence of neurolathyrism in Dawunt district using prospective studies; and should examine the association between neurolathyrism and social and environmental factors.

### Conclusion and recommendations

In Dawunt woreda, the prevalence of neurolathyrism was found to be high. Age, Sex, Marital status, Family size, Annual grass pea production amount, Ever feeding only grass pea, Grass pea to other cereals mixing ratio, and Ever feeding immature grass pea seeds were found to have statistically significant association with an increased odds of neurolathyrism.

Therefore, it is better if residents of Dawunt district reduce the consumption of grass pea or if they consume it by mixing with other cereals using a ratio of less than 1:1. They should also avoid consuming grass pea without mixing with other cereals. Moreover, they should avoid consuming immature seeds of the grass pea. It is also better if health care workers provide health education on the possible methods for reducing the toxic effects of grass pea for residents of Dawunt district. It is also better if administrators of Dawunt woreda and the government together provide alternative non-toxic grass pea seeds for farmers in Dawunt district. With administrators of Dawunt district and the government, it is also better if NGOs and project designers propose and implement a project intended to support patients with neurolathyrism. It is also better if future researchers conduct further studies by addressing the limitations of this study.

## Data Availability

All data generated or analysed during this study are included in this published article.
